# Notes on Preparations for the Sick

**Published:** 1910-12

**Authors:** 


### Notes on Preparations for the Sick.

**Ceregen: a proteid-phosphate food powder.**—JOHN BELL, HILLS & LUCAS Ltd., London, S.E.—Is tasteless, may be added to any food, or one to four teaspoonfuls may be dissolved in warm water; is best taken with meals, and is a food, not a medicine.

Ceregen should prove to be a valuable addition to the usual articles of diet in cases where extra nitrogenous matter in a readily-digestible form is required.

Our examination confirms the statements of the makers as regards its composition and the total absence of starch.

---

**Albulactin.**—A. WULFING & Co., Chenies Street, W.C.—Albulactin is guaranteed by the makers to be a pure, soluble, milk-albumin. It is recommended as an adjunct to cow's milk, and formulæ are provided by means of which "humanised" milk may be readily prepared.

Numerous analyses have been made, and there is little doubt as to the identity of the substance with "Lact-albumin." Our own examination showed the substance to consist of soluble proteid. The clinical reports are also most satisfactory, and these bear more weight than chemical examinations.

---

**"Lactitia."**—NUTRICIA LTD., Loetermeen, Holland.—This is a preparation of condensed Dutch buttermilk with the addition of flour, and is supplied in hermetically sealed tins. We have made a chemical analysis, and find that it contains approximately 40 per cent. of total solids. The small amount of fat which is present rather detracts from its value as a food.

---

**Biogène Diabetic Biscuits.**—BONTHRON & Co., 106 Regent Street, London, W.—Biogène biscuits are one of a series of specialities made by this firm, their basis being a soluble casein with the addition of fatty and mineral matters.

They are not unpleasant to the taste, and chemical analysis failed to reveal the presence of starch—a significant fact, inasmuch as starch is frequently present in quantity in the so-called "starch-free" preparations for the diabetic.

---

**Hordine: Liquid Malt.**—OPPENHEIMER, SON & Co., London.—A food in itself, and it assists the digestion of ordinary starchy foods. One to four teaspoonfuls by itself, or with aerated water or milk, should be taken directly after meals.

It is a liquid preparation obtained from selected malted barley, and one which lacks the excessive sweetness of many of the semi-solid extracts.

The essential feature of a malt extract should be the presence in it of an active amylolytic ferment. We have examined Hordine from this point of view, and we find that it is capable of digesting starch at the ordinary body temperature. It can therefore be recommended to those who need a proteid containing food with digestive properties.

**Thiocol.** HOFFMANN, LA ROCHE & Co., London.—The potassium salt of guaiacol-sulphonic acid, containing 52 per cent. of guaiacol, is a fine powder, odourless, with a slightly bitter taste, readily soluble in water. It may be given in daily doses of two to four drachms, and it has none of the disadvantages of creosote and guaiacol.

Although drugs are not much in favour with the physicians who are carrying on sanatorium treatment, it is well to bear in mind that there is much evidence in favour of the utility of this drug, and that there are practically no drawbacks attending its use. It is conveniently put up in tablets of five grains each, but for larger doses the powder form must be more suitable.

---

"**Perhydrol.**"—E. MERCK (16 Jewry Street, E.C.), Darmstadt.—This preparation is a one hundred volume solution of hydrogen peroxide (30 per cent. by weight), and is thus ten times stronger than the P.B. hydrogen peroxide. Concentrated hydrogen peroxide solutions are somewhat unstable, and this fact has hitherto stood in the way of their use, but by packing the solution in bottles lined with a layer of paraffin wax, and using a cork of the same material, this difficulty has been successfully overcome. Thus some weeks after the bottle had been opened the preparation was found to be still active. By adding to the Perhydrol nine volumes of water the ten-volume solution commonly used can be prepared. The undiluted preparation has a slight caustic action. Peroxide of hydrogen is now used so largely, both in general and special surgery as a hæmostatic, disinfectant and deodorant, that any description of its uses is needless. This preparation can be recommended as being acid free, constant and convenient.

By an ingenious arrangement in the cork the solution can be either dropped or poured out as required.

---

**A New Antiseptic Dressing.**—BURROUGHS, WELLCOME & Co., London.—**Tabloid Brand Bismuth Gauze** is a new dressing which will be particularly acceptable to the gynæcologist, and also in surgical cases generally. This preparation is non-toxic and has no smell. Its properties are equivalent in other respects to those of iodoform gauze. Placed as an absorbent dressing in the uterine cavity Bismuth Gauze has remained odourless for five days, whereas iodoform gauze under similar circumstances became foul in forty-eight hours.

Bismuth Gauze would appear, therefore, to be specially suitable for use in cases of incomplete abortion ; in curretting, plastic operations on the cervix and vagina, and for many other purposes where a powerful non-irritating antiseptic is required. It is made in widths of one, two and three inches, and is carefully sterilised and packed in one-yard rolls each surrounded by a specially varnished germ-proof cover.

---

**Sinapisms: improved mustard plasters.**—J. and J. Colman, London.—The valuable properties of pure mustard as a safe and rapidly-acting rubefacient are so well known as to render it unnecessary to insist upon them, and in the preparation of this improved plaster nothing but pure mustard is used.

The necessity to the household of having some reliable preparation of mustard as a counter-irritant always at hand, ready for immediate application is self-evident; and Colman's Sinapism possesses many advantages.

1. It is quickly and easily applied, it being necessary simply to immerse the plaster in water for one second and it is ready for immediate application.
2. It is cleanly, the mustard not coming into direct contact with the skin, and consequently not adhering when the plaster is taken off.
3. It is very prompt and certain in its effect, commencing its action almost immediately it is applied.
4. It takes up more moisture, and consequently continues its action.
5. The mustard is spread on a thin pliable fabric, and therefore the Sinapism accommodates itself to the various parts of the body.
6. It is conveniently portable, either in a pocket-book, travelling case, etc.
7. It is free from any unpleasant smell.

---

**Syrup Cocillana Compound.** Parke, Davis & Co., London. —Each fluid ounce contains:—

| | |
|---|---|
| Heroin Hydrochloride .. .. .. .. | gr. $\frac{8}{24}$ |
| Træ Euphorb. Piluliferæ .. .. .. | ♏ 120 |
| Syrup Wild Lettuce .. .. .. .. | ♏ 120 |
| Tinct. Cocillanæ .. .. .. .. .. | ♏ 40 |
| Syrup Squill. Comp. U.S.P. .. .. .. | ♏ 24 |
| Cascarin (P. D. & Co.) .. .. .. .. | gr. 8 |
| Menthol .. .. .. .. .. .. .. | gr. $\frac{8}{100}$ |

This elaborate compound may be given in doses of one drachm or more in cases where the component drugs are indicated, but we think that the heroin is by far the most active of the components, and must give the more innocent ingredients a chance of getting credit from being in good company.

